# Usefulness of Endoscopic Ultrasound Strain Elastography for Measuring Liver Stiffness and the Role of Blood Cytokeratin 18 Levels as a Surrogate Marker of Fibrosis

**DOI:** 10.5152/tjg.2025.24070

**Published:** 2025-06-16

**Authors:** Deniz Guney Duman, Yesim Ozen Alahdab, Coskun Ozer Demirtas, Yusuf Yılmaz, Feyza Dilber, Filiz Ture Ozdemir, Caglayan Keklikkiran, Haluk Tarik Kani, Umut Emre Aykut, Osman Cavit Ozdogan

**Affiliations:** 1 Division of Gastroenterology and Hepatology, Marmara University School of Medicine, İstanbul, Türkiye; 2 Institute of Gastroenterology, Marmara University, İstanbul, Türkiye; 3 Department of Gastroenterology, Recep Tayyip Erdoğan University School of Medicine, Rize, Türkiye; 4 Division of Hematology and Immunology, Marmara University School of Medicine, İstanbul, Türkiye

**Keywords:** Chronic liver disease, cirrhosis, elastography, endoscopic ultrasonography, liver stiffness, strain ratio

## Abstract

**Background/Aims:**

The role of semi-quantitative strain ratio (SR) using real-time endoscopic ultrasound strain elastography (EUS-E) in chronic liver disease (CLD) and cirrhosis is yet to be determined. Herein, the aim was to assess the usefulness of EUS-E to detect CLD and cirrhosis.

**Materials and Methods:**

Patients with cirrhosis and non-cirrhotic CLD were enrolled prospectively. Patients without liver disease and undergoing EUS examinations for non-hepatic indications were taken as control group. Strain ratio was calculated from strains of hepatic vein and liver parenchyma. Fibrosis-4 (FIB-4) and aspartate aminotransferase (AST)-to-Platelet Ratio Index (APRI) scores were recorded, and blood cytokeratin-18 (CK-18) levels were measured to assess hepatic fibrosis. A clinical evaluation was also conducted.

**Results:**

One hundred participants (control: 49, CLD: 33, cirrhotic: 18) were included. The SR and liver parenchyma strains in cirrhotics were significantly higher than those in the CLD (*P* < .001) and control (*P* < .001) groups. Strain ratio threshold set at 5.67 had a sensitivity of 94.4% and a specificity of 95.9% to differentiate cirrhotics from control patients. An SR threshold of 10.65 had a sensitivity of 94.4% and a specificity of 84.8% in differentiating cirrhotics from CLD patients. The SR showed a strong positive correlation with FIB-4 and APRI scores, but not with CK-18 levels.

**Conclusions:**

Strain ratio thresholds of 5.67 and 10.65 obtained by EUS-E are useful to differentiate cirrhotics from non-cirrhotic CLD patients and liver-disease-free subjects, respectively. This pilot study is the first one evaluating the role of EUS-E in liver diseases, and future studies involving patients having CLD of specific etiologies are warranted.

**Main Points**

- To date, the semi-quantitative SR measured by EUS-E has not been investigated for differentiating cirrhosis from non-cirrhotic CLD and healthy controls.
- Endoscopic ultrasound strain elastography guided SR threshold of 5.67 differentiated cirrhotic from healthy controls with high sensitivity (94.4%) and specificity (95.9%), while an SR threshold of 10.65 was effective in distinguishing cirrhotic from non-cirrhotic CLD patients (sensitivity: 94.4%, specificity: 84.8%).
- Endoscopic ultrasound strain elastography guided SR strongly correlated with FIB-4 and APRI scores, indicating its potential as a minimally invasive marker of liver fibrosis.
- Further studies are required to validate SR thresholds in patients with CLD of specific etiologies.

## Introduction

Differentiating cirrhosis is vital for the management of chronic hepatitis B virus (HBV), chronic hepatitis C virus (HCV), and non-alcoholic steatohepatitis (NASH), which are the 3 most common etiologies for chronic liver disease (CLD).^1^ Viral hepatitis remains the main cause of cirrhosis, HCC, and liver transplantation in Türkiye.^2^^-^^4^ However, its prevalence seems to be declining, whereas the prevalence of steatotic liver disease-related cirrhosis is increasing. Identification of liver cirrhosis is the most important cornerstone for the management of CLD, which influences the surveillance strategies as well. Liver biopsy for diffuse liver disease is now believed not to be the gold standard but maybe the silver standard because a limited number of portal areas in a tiny liver biopsy cannot represent the whole organ. Additional limiting factors of biopsy are its invasiveness and considerable inter- and intrao-bserver variability among different pathologists.^5^ Transient elastography, namely FibroScan, on the other hand, uses the velocity of ultrasound waves in solid organs, and the shear wave elasticity probe quantifies the “stiffness” of hepatic parenchyma and correlates that with the stage of liver disease.^6^ In stiff and fibrotic tissue, the ultrasound wave moves more swiftly. In fact, it is well known that liver stiffness is related to the degree of hepatic fibrosis and, till now, a significant amount of data has been published to incorporate its use in daily practice of liver fibrosis assessment.^7^ Despite being a noninvasive method, the presence of ascites, obesity, advanced liver tissue atrophy, and narrow intercostal spaces are listed as the limitations of transient elastography.

To screen advanced fibrosis in patients with CLD, numerous compound surrogates—based on routine clinical and laboratory parameters—have been developed, of which the globally acknowledged ones are Fibrosis-4 (FIB-4) and aspartate aminotransferase (AST)-to-Platelet Ratio Index (APRI) scores. However, these simple non-invasive scores are mainly useful to exclude, but not to predict, advanced fibrosis in the clinic.^8^ Cytokeratin 18 (CK-18), a keratin-containing protein found in the cytoskeletal architecture of epithelial and parenchymal cells, is a candidate non-invasive biomarker for apoptosis, liver disease activity, and fibrosis assessment in CLD.^9^^,10^ It is abundant in hepatocytes, and CK-18 filaments are cleaved by caspases during hepatic necrosis and apoptosis; thus, M30 fragments are among the resulting products of this caspase cleavage. Owing to its rich perfusion into the liver, CK-18 M30 fragments can be easily detected in the systemic circulation. In clinical studies, serum CK-18 has been shown to be related strongly to the development and progression of liver fibrosis demonstrated by transient elastography and liver biopsy.^11^^-^^13^

Endoscopic ultrasound strain (EUS) has a wide range of indications, such as pancreatic masses, subepithelial gastrointestinal masses, and diseases involving the biliary tract and mediastinum. EUS devices have special software for elastography to measure liver stiffness, making it possible to check real-time liver stiffness in patients undergoing EUS examination for a variety of the above indications. Nevertheless, unlike transient elastography, the potential of EUS to measure liver stiffness is yet to be defined.^14^ Strain ratio (SR) is simply the comparison of the strain of a lesion to normal reference tissue within the same region of interest (ROI). Semi-quantitative SR using real-time EUS elastography (EUS-E) has been studied extensively for focal pancreatic masses and lymph nodes, but its role in CLD is yet to be determined.

To date, the usefulness of EUS‑E for the assessment of liver fibrosis has not been studied. The aim was to assess the role of EUS-E using semi-quantitative (strain ratio) measures for differentiating advanced liver fibrosis from mild liver damage. The non-invasive fibrosis indicators were also incorporated into this study, including FIB-4, APRI score, and serum CK-18 values to support the liver fibrosis assessment.

## Materials And Methods

### Patient Selection and Data Collection

Cirrhotic and non-cirrhotic CLD patients, including etiologies of HBV, HCV, and NASH patients who were referred to the EUS clinic for indications such as evaluation of submucosal lesions, presence of submucosal varices, or suspicion of any biliary stone disease, were enrolled prospectively between 2017 and 2019. Cirrhosis was diagnosed clinically and by radiologic imaging or liver biopsy. Patients without any sign of liver disease and who had undergone EUS examinations for non-hepatic indications, such as the assessment of subepithelial masses, were taken as a control group. All patients underwent EUS-E procedures, and all measurements were noted. Exclusion criteria included the presence of malignancies, biliary stricture, or other benign causes that may affect the biliary system, and the inability to complete the EUS procedure.

For all participants, laboratory parameters (AST, alanine aminotransferase (ALT), and platelet count were determined at the time of the EUS examination. Aspartate aminotransferase to platelet ratio index score [AST (IU/L) / Platelet count] and FIB-4 score [Age (year) × AST (IU/L)] / [Platelet (10^9^/L) × (sqr (ALT) (IU/L)] were calculated using clinical and laboratory data exactly as described.^15^^,^^16^

Peripheral venous blood for serum CK-18 M30 measurement was obtained from all subjects at the same setting of EUS and was stored at −80°C until assayed.

### The Quantitative Determination of the Apoptosis-Associated Caspase-Cleaved Keratin 18

Blood samples from patients and healthy controls were centrifuged at 3000 × g for 5 minutes, and serum aliquots were stored directly at −80°C until used for the assay. Serum levels of CK18-Asp396 (M30) were determined by a commercially available 1-step in vitro immunoassay (M30-Apoptosense ELISA kit, Peviva AB, Bromma, Sweden) according to the manufacturer’s protocol. In brief, 25 µL of M30 Standard (A–G), M30 Control Low, M30 Control High, or samples were placed into wells, and then 75 µL of the diluted M30 Conjugate solution were added to each well. The wells were coated with mouse monoclonal K18 antibody M5. After 4 hours of incubation, the plate was washed with wash solution 4 times and then 200 µL of TMB Substrate were added to each well. After 20 minutes of incubation in darkness, stop solution were added to each well, and the plate was read by an ELISA reader at 450 nm. By plotting a standard curve from known concentrations versus measured absorbance, the amount of antigen in the sample was calculated. The concentration of the antigen was expressed as units per liter (U/L).

### Strain Measurement Using Endoscopic Ultrasound Strain Elastography

**Figure 1.**
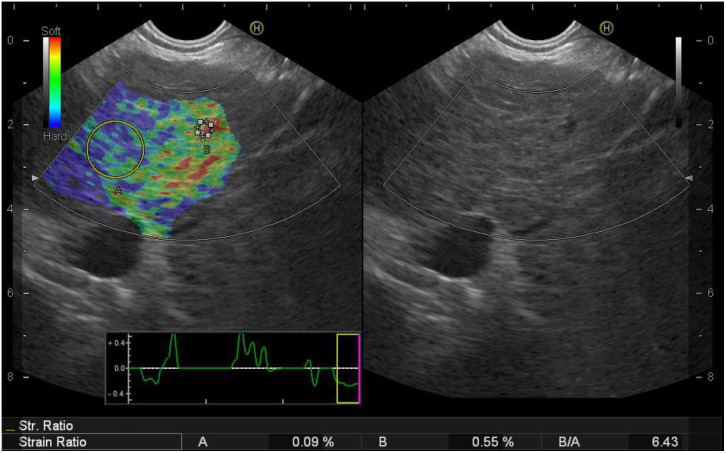
Strain measurement using endoscopic ultrasound strain elastography.

Strains from a sufficient size of ROI from the left liver lobe parenchyma (A) in reference to a hepatic vein area (B) were measured from the same frozen image, and SR were automatically calculated by the US device as the division of B/A.^17^ Small intrahepatic veins and their walls in the same frozen image were chosen because the strain is uniformly distributed in all structures. Unlike portal vein branches, intrahepatic veins are not exposed to liver stiffness due to portal hypertension or atherosclerosis. The area in immediate proximity to the small hepatic vein wall was chosen as a control parameter. At least 3 sets of measurements were obtained from each patient, and their average was used for the SR. Strain elastograms were generated by the internal physiological pulsations from cardiac or respiratory contractions and detected by holding the transducer still in a suitable position. The strain image is displayed as a color image with hard tissue visualized by blue, indicating minor strain and soft tissue visualized by red (Figure 1).

### Ethical Statement

The study protocol was approved by Marmara University Medical School Ethics Committee (December 2, 2016, numbered: 09.2016.592). It is prospectively registered to Australia New Zealand Clinical Trials Registry (March 27, 2017). The study was conducted in compliance with good clinical practice and the principles of the Declaration of Helsinki. All patients gave written consent to participate after having received full information about the study.

### Statistical Analysis

All continuous variables were assessed for normality distribution using Shapiro–Wilk and Kolmogorov-Smirnov tests and reported as median and interquartile range or mean ± SD when appropriate. Categorical variables were given as frequency and percentage. For the comparison of continuous variables among 3 groups, a one-way ANOVA test with post-hoc LSD test was used when the data conformed to a normal distribution; otherwise, the Kruskal–Wallis test with post-hoc Mann-Whitney U test was used. Correlation analyses were performed by Spearman’s bivariate correlation test. To determine the diagnostic accuracy, receiver operating characteristic (ROC) curves were drawn and the area under the curve (AUC) was calculated. The optimal cut-off values were identified by calculating the Youden index. Sensitivity, specificity, positive and negative predictive values, and likelihood ratios were calculated with their CI on a per-patient basis. *P*-values < .05 were considered statistically significant. Statistical analyses were performed using SPSS software version 20.0 (IBM SPSS Corp.; Armonk, NY, USA).

## Results

Baseline demographic, laboratory, and EUS-E results of the studied groups are presented in Table 1 with the post-hoc comparisons of statistically significant differences. A total of 100 participants (cirrhotic: 18, non-cirrhotic CLD: 33, control: 49) were enrolled. The SR in cirrhotics (26.96 (4.19-216.05)) was significantly higher than in the chronic hepatitis (*P* < .001) and control (*P* < .001) groups. Average liver parenchyma strain in cirrhotics (0.083 ± 0.0600) was significantly lower than in the CLD (*P* < .001) and control (*P* < .001) groups, while the CLD group also had a statistically significant lower value in comparison to controls (*P* < .001). No difference was observed between the 3 groups with regard to hepatic vein strains. Aspartate aminotransferase to platelet ratio index score, FIB-4 score, and CK18 values, as non-invasive liver fibrosis indicators, increased significantly from controls to CLD and cirrhosis (all 3 *P*-values <.05).

**Figure 2.**
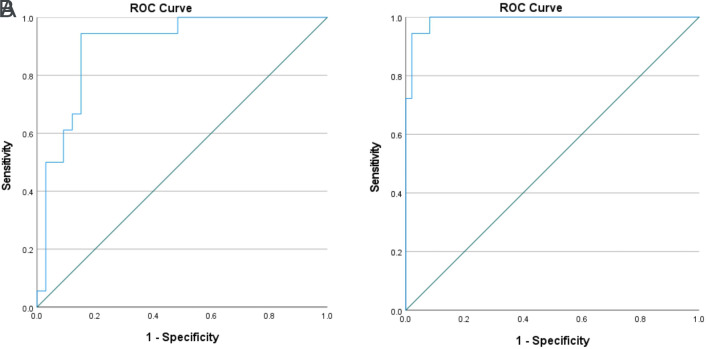
Receiver operator curves for endoscopic ultrasound strain-elastography strain ratio to differentiate cirrhotics from chronic liver disease (A) and healthy controls (B).

The performance of EUS-elastography liver SR to differentiate cirrhotics from CLD and controls is given in Table 2. The threshold value for SR to differentiate cirrhotics from control patients set at 5.67 obtained from ROC curve analysis had a sensitivity of 94.4% and a specificity of 95.9% with an AUC value of 0.991 (Figure 2A). The optimal cut-off value of 10.65 for SR had a sensitivity of 94.4% and a specificity of 84.8% with an AUC value of 0.901 in differentiating cirrhotics from non-cirrhotic CLD patients (Figure 2B).

Liver SR positively correlated with APRI (r = 0.474, *P* < .001) and FIB-4 (r = 0.382, *P* < .001), while the correlation analysis of CK-18 and liver SR did not reach statistical significance despite a positive tendency (r = 0.206, *P* = .063) (Table 3).

## Discussion

Currently, the role of EUS-guided elastography in identifying liver fibrosis is controversial. In this clinical pilot study, whether SR obtained by EUS-E could distinguish the presence of cirrhosis was assessed. The results have shown that SR, obtained by EUS-E, can differentiate cirrhotic patients from non-cirrhotic CLD patients and healthy controls. The optimal EUS-E SR cut-off values were searched to differentiate cirrhosis and found to be 10.65 for CLD patients and 5.67 for healthy controls, and both estimates showed very good diagnostic and discriminative performance. The strong positive correlation of SR with FIB-4 and APRI supported its potential role in fibrosis assessment.

Herein, a new method for the calculation of SR is described rather than merely using strain. Strain ratio, although expressed numerically, is considered to be a semi-quantitative measurement because the strain in the ROI is defined in reference to surrounding tissue which is assumed to be normal and uniform. Therefore, the blood vessel wall is chosen as the reference tissue. It was believed that using SR instead of strain-only measurements enables us to obtain repeatable and better-standardized results despite being semi-quantitative. Obviously, new studies evaluating the efficacy of this method are warranted.

The introduction of new transabdominal ultrasound devices with elastography functions has raised the interest in using them for liver fibrosis assessment. Nevertheless, there is no established consensus for the correct application method of elastography. Fang et al^18^ have assessed the role of real-time strain elastography on transabdominal ultrasound and compared their results with liver biopsies obtained immediately after the strain image acquisitions. The main difference between their study and this one is they did not compare the strain with any normal tissue, thus no SR was generated. The SR method that has been described can certainly be applied to transabdominal liver elastography. Nevertheless, EUS‑E assessment of liver fibrosis is not expected to replace transabdominal transient elastography because of its invasive nature and higher cost, but it may be a promising tool to complete the examination in patients without apparent biliary tract conditions and who are already undergoing an EUS examination for another indication. An additional advantage of EUS-E is the possibility of obtaining a liver biopsy during the procedure when needed.

Surveillance in CLD is recommended to screen for liver fibrosis and identify patients who develop cirrhosis or advanced fibrosis. Liver biopsy is considered the gold standard for detecting liver fibrosis; however, it carries serious limitations, including invasiveness, high cost, risk of rare but potentially life-threatening complications, and obtaining insufficient material.^19^^-^^21^ To overcome those limitations of liver biopsy, new methods are under investigation. Among serum fibrosis models, APRI and FIB-4 scores are commonly used to identify liver fibrosis and cirrhosis in patients with CLD. Both have successfully predicted liver fibrosis in large cohorts of patients with CLD.^16^^,22^ The cut-offs of APRI (>1.50) and FIB-4 (>3.25) are proposed for the diagnosis of advanced fibrosis with high sensitivity, reaching up to 90%-95%.^15^^,23^ Both serum markers have been integrated into this study and were found to correlate well with EUS-E SR values in this cohort.

The aim was to enrich the study by testing serum CK-18 levels and their correlation with EUS-E SR results. CK-18 is a potential non-invasive liver fibrosis indicator and several studies have reported an association of it with the stage of liver fibrosis in CLD due to etiologies of HBV, HCV, and nonalcoholic fatty liver disease (NAFLD).^24^^-^^26^ Among HCV patients infected mainly with genotypes 1 and 3, CK-18 levels of >330 U/L had distinguished mild-moderate fibrosis from advanced fibrosis with a sensitivity and specificity of 89% and 78%, respectively.^25^ Another study in HBV patients to determine the diagnostic potential of CK-18 showed a good discriminatory ratio for patients with moderate/severe versus mild fibrosis (AUC: 0.86, *P* < .0001), with 84% sensitivity and 80% specificity for a CK-18 value of 253 IU/mL.^26^ When NAFLD patients were studied, a CK-18 cut-off value of 375 U/L was effective in differentiating the NAFLD activity score (NAS) >5 patients from NAS< 4 patients with a sensitivity of 81.5% and specificity of 65% (AUC of 0.79, *P* < .001).^27^ In this study, a correlation tendency between the levels of CK-18 and EUS-E SR results was found, but it was not statistically significant.

With the advancement of endohepatology, new linear echoendoscopes that can perform EUS-guided shear wave elastography have been produced. Although there is no data comparing EUS-guided strain elastography with EUS-guided shear wave elastography for hepatic lesions, there is 1 study assessing the diagnostic performance of endoscopic ultrasonography-guided shear-wave measurements versus strain elastography for solid pancreatic lesions.^26^ In this comparative study, conventional strain elastogram with strain histogram was found to be superior in its characterization capacity, differentiating pancreatic cancer from mass-forming pancreatitis, while EUS-guided shear wave could not differentiate malignant from benign lesions.^26^ Nevertheless, 1 recent study compared EUS-guided shear wave elastography with transabdominal transient elastography using shear waves in diffuse hepatic disease.^26^ The diagnostic accuracy of EUS-guided shear wave elastography correlated well with liver histology, and liver fibrosis assessment using EUS shear wave elastography was comparable with transabdominal transient elastography.^26^ In this study, strain elastogram of liver parenchyma and strain ratio were used with the vascular wall structure, which is a valuable fibrosis assessment method.

Limitations of this study were as follows: a) Smaller reference areas of hepatic veins were used in the surrounding liver tissue, and this approach may be prone to artifacts. b) For standardization purposes, left liver lobe measurements were obtained in all cases whereas liver biopsies and transient elastography use the right liver lobe. Although EUS can also visualize the right liver lobe, the hypertrophied left liver lobe assessment, especially in cirrhotics, may bring new insight to hepatology. c) Liver biopsies from these cases were not obtained for ethical reasons. Therefore, there is a small possibility that a few of the CLD patients might have had advanced fibrosis without signs of cirrhosis, and liver biopsy before EUS could have clarified any uncertainty regarding this point. However, all of the cirrhotic patients were confirmed clinically, radiologically, and through laboratory tests. Those with chronic viral hepatitis or NAFLD, without any suspicion of cirrhosis, were categorized as having CLD. The descriptive study may pave the way for new studies harboring patients with different fibrosis grades proven via liver biopsy. d) Applied stress by the internal pulsations to generate the strain is considered to be distributed evenly, which may not be the case arithmetically. To minimize this bias, the nearest reference to the target tissue was selected, assuming both areas are exposed to a similar quantity of stress.

In conclusion, despite being an invasive procedure, SR thresholds of 5.67 and 10.65 obtained through EUS-E are useful for differentiating cirrhotic patients from non-cirrhotic CLD patients and healthy controls, respectively. It has been demonstrated that SR values correlate well with the serological hepatic fibrosis markers APRI and FIB-4, although the correlation with CK-18, another potential serum surrogate marker for fibrosis, was poor. This pilot study is the first to evaluate the role of EUS-E in liver cirrhosis, and future studies involving CLD patients with varying levels of liver fibrosis and different specific etiologies are warranted.

## Figures and Tables

**Table 1.** Baseline Demographics and Endoscopic Ultrasound Strain-Elastography Characteristics

	Controls (n = 49)	CLD (n = 33)	Cirrhosis (n = 18)	*P*
Age*,* years	51.2 ± 15.2	54.2 ± 12.0	61.6 ± 9.6	.020* (CON vs CLD: .061) (CON vs CIR: 0.061) (CLD vs CIR: .005*)
Sex Male Female	19 (38.8) 30 (61.2)	15 (45.5) 18 (54.5)	8 (44.4) 10 (55.6)	.813
Liver disease etiology Viral NAFLD	-	9 (27.3) 24 (72.7)	8 (44.4) 10 (55.5)	.233
AST*,* IU/L	20 (11-38)	23 (13-118)	35 (15-186)	.001* (CON vs CLD: .007*) (CON vs CIR: .048*) (CLD vs CIR: .719)
ALT*,* IU/L	18 (7-61)	25 (9-222)	22 (11-168)	.014* (CON vs CLD: .007*) (CON vs CIR: .048*) (CLD vs CIR: .719)
Platelet count, med (min-max)*,* ×1000/m^3^	246 (154-388)	212 (150-659	80 (44-289)	<.001* (CON vs CLD: .348) (CON vs CIR:<.001*) (CLD vs CIR:<.001*)
APRI score	0.19 (0.10-0.56)	0.26 (0.08-1.08)	0.90 (0.13-8.45)	<.001* (CON vs CLD: .034*) (CON vs CIR:<.001*) (CLD vs CIR:<.001*)
FIB-4 score	1.05 (0.31-3.28)	1.14 (0.32-2.34)	5.27 (0.66-21.38)	<.001* (CON vs CLD: .317) (CON vs CIR: <.001*) (CLD vs CIR: <.001*)
CK-18, mg/dL	142 (0.1-743.0)	192.0 (46.8-887.0)	214.0 (28.4-977)	.035* (CON vs CLD: .043*) (CON vs CIR: .030*) (CLD vs CIR: .588)
Average liver strain	0.4563 ± 0.1715	0.3106 ± 0.1885	0.083 ± 0.0600	<.001* (CON vs CLD: <.001*) (CON vs CIR: <.001*) (CLD vs CIR: <.001*)
Average vein strain	1.0529 ± 0.3776	1.1094 ± 0.4897	1.0683 ± 0.5176	0.850
Liver strain ratio	2.23 (0.93-13.21)	3.94 (0.96-207.14)	26.96 (4.19-216.05)	<.001* (CON vs CLD: <.001*) (CON vs CIR: <.001*) (CLD vs CIR: <.001*)

AST, aspartate aminotransferase; ALT, alanine aminotransferase; APRI, AST to platelet ratio index; CIR, cirrhosis group; CK-18, cytokeratin-18; CLD, chronic liver disease; CON, control group; EUS, endoscopic ultrasonography; FIB-4, fibrosis-4; HBV, hepatitis B virus; HCV, hepatitis C virus; NAFLD, nonalcoholic fatty liver disease.

*Indicates statistically significant difference among groups.

**Table 2.** Performance of Endoscopic Ultrasound Strain-Elastography Liver Strain Ratio to Differentiate Cirrhotics

	vs. Controls	vs. CLD
Optimal cut-off value	5.67	10.65
Sensitivity, % (95% CI)	94.4 (72.7-99.9)	94.4 (72.7-99.9)
Specificity, % (95% CI)	95.9 (86-99.5)	84.9 (68.1-94.9)
PPV, % (95% CI)	89.5 (68.5-97.1)	77.3 (60.1-88.5)
NPV, % (95% CI)	97.9 (87.5-99.7)	96.6 (80.6-99.5)
LR (+), % (95% CI)	23.1 (5.9-90.3)	6.2 (2.8-14.1)
LR (-), % (95% CI)	0.06 (0.01-0.39)	0.07 (0.01-0.44)
Accuracy, % (95% CI)	95.5 (87.4-99.1)	88.2 (76.1-95.6)
AUC, % (95% CI)	0.991 (0.975-1.000)	0.901 (0.814-0.988)

AUC, area under the curve; CLD, chronic liver disease; LR (+), positive likelihood ratio; LR (−), negative likelihood ratio; NPV, negative predictive value; PPV, positive predictive value.

**Table 3.** Spearman Correlation Analysis Among Aspartate Aminotransferase to Platelet Ratio Index, Fibrosis-4, Cytokeratin-18, and Liver Strain Ratio

	Whole Cohort (n = 100)
APRI – Liver strain ratio	r = 0.474 *P* < .001*
FIB-4 – Liver strain ratio	r = 0.382 *P* < .001*
CK-18 – Liver strain ratio	r = 0.206 *P* = .063

APRI, aspartate aminotransferase to platelet ratio index; FIB-4, fibrosis-4; CK-18, cytokeratin-18.

*Indicates statistically significant *P*-values.

## Data Availability

All data for this article will be available upon request.
